# TREYESCAN: configuration of an eye tracking test for the measurement of compensatory eye movements in patients with visual field defects

**DOI:** 10.1038/s41598-023-47470-5

**Published:** 2023-11-22

**Authors:** Yasmin Faraji, Joris W. van Rijn, Ruth M. A. van Nispen, Ger H. M. B. van Rens, Bart J. M. Melis-Dankers, Jan Koopman, Laurentius J. van Rijn

**Affiliations:** 1 Department of Ophthalmology, Amsterdam UMC Location Vrije Universiteit Amsterdam, Amsterdam, The Netherlands; 2 Amsterdam Public Health, Quality of Care, Societal Participation & Health, Mental Health, Aging and Later Life, Amsterdam, The Netherlands; 3 Royal Dutch Visio, Centre of Expertise for Blind and Partially Sighted People, Huizen, The Netherlands; 4 Department of Ophthalmology, Onze Lieve Vrouwe Gasthuis, Amsterdam, The Netherlands; 5 Amsterdam Neuroscience, Systems & Network Neurosciences, Amsterdam, The Netherlands

**Keywords:** Glaucoma, Quality of life

## Abstract

The Traffic Eye Scanning and Compensation Analyzer (TREYESCAN) is introduced as an innovative eye tracking test designed to measure compensatory eye movements in individuals with visual field defects. The primary objective of the test is to quantitatively assess and analyze the compensatory eye movements employed by patients with visual field defects while viewing videos of various traffic scenes from the viewpoint of a driver of a passenger car. The filming process involved capturing a wide range of driving conditions and hazards, aiming to replicate real-world scenarios. Specific dynamic areas of interest within these scenes were selected and assessed by a panel of experts on medical and practical fitness to drive. Pilot measurements were conducted on a sample of 20 normally-sighted individuals during two different measurement sessions. The results provide valuable insights into how individuals without visual impairment view the dynamic scenes presented in the test. Moving forward, the TREYESCAN will be used in a case–control study involving glaucoma patients and control subjects, with the goal of further investigating and understanding the mechanisms employed by individuals with glaucoma to compensate for their visual field defects.

## Introduction

Primary open-angle glaucoma (POAG) is the leading cause of irreversible blindness in the world^1^, characterized by progressive damage to the optic nerve and consequent visual field loss^2^. The impact of this condition significantly affects the daily lives and independence of those affected^3,4^. Driving and mobility, in particular, are crucial aspects that contribute to an individual's quality of life and sense of autonomy^5^. The current method of static visual field testing does not properly discriminate between persons with visual field defects that are fit and unfit to drive^6^. Merely testing visual field defects does not reflect the impairment of those affected since defects may be compensated for, such as by head and eye movements.

While previous studies have explored compensatory eye movements in persons with glaucoma during on-road driving^7,8^, driving simulator tests^9,10^, and hazard perception tests^11,12^, a knowledge gap remains regarding the mechanisms by which these patients use head and eye movements to compensate for their visual field defects. This is particularly relevant in the context of dynamic areas of interest (AOIs) within traffic scenes, which refer to specific regions of a stimulus that are relevant for the research objective. AOIs are utilized to measure various metrics such as AOI hits, which occur when gaze coordinates fall within an AOI^13^. The analysis of dynamic AOIs, which refer to moving regions of interest that emerge during video presentations or animated elements on a screen, presents a challenge due to the movement of these objects relative to the coordinate system in which the gaze position data is recorded^14^. Existing research has primarily focused on fixations and saccades, without exploring the relevance of viewing AOIs. Investigating AOI hits, entry times and dwell times can improve the analysis of compensatory eye movements. For instance, establishing a correlation between the frequency of saccades and AOI hits, could shed light on the quality of compensatory eye movements in persons with visual field loss. Exploring these compensatory mechanisms is of importance, as they play a crucial role in helping individuals with visual field defects in overcoming visual limitations and maintaining functional performance in visually demanding situations, like driving.

To bridge this research gap, we have developed an innovative eye tracking test, the TREYESCAN (Traffic Eye Scanning and Compensation Analyzer), with the objective of measuring compensatory eye movements in patients with visual field defects. The TREYESCAN measures eye movements over a wide field of view (100°), recognizing the vital role played by the visual field in safe traffic participation^15^. Given that visual field defects may reside in the periphery, it is apparent that eye movements need to be assessed on a large screen, rather than solely focusing on a small centrally located monitor, covering approximately 30º. Additionally, transportation research has highlighted that a restricted field of view of driving scenes (presented on a single monitor) can lead to poorer hazard detection and less eccentric eye movements compared to setups involving additional side views on adjacent monitors^16,17^. Consequently, we developed an accessible method for analyzing eye movements on a screen with a wide field of view (100°), while accommodating unrestricted head and eye movements.

This study introduces the methodology employed in the development of the TREYESCAN. We provide insights into the process of traffic scene selection and identification of areas of interest within the videos. We carefully curated a diverse range of traffic scenarios, encompassing various driving conditions and potential hazards. Additionally, we investigate the results obtained from pilot measurements conducted on a group of normally sighted individuals. By examining the gaze behavior and eye movements of individuals with normal vision, we gain a baseline understanding of how individuals without visual field defects interact visually with the dynamic scenes presented in our test.

## Methods

### Experiment setup, recording device and analysis software

**Figure 1:**
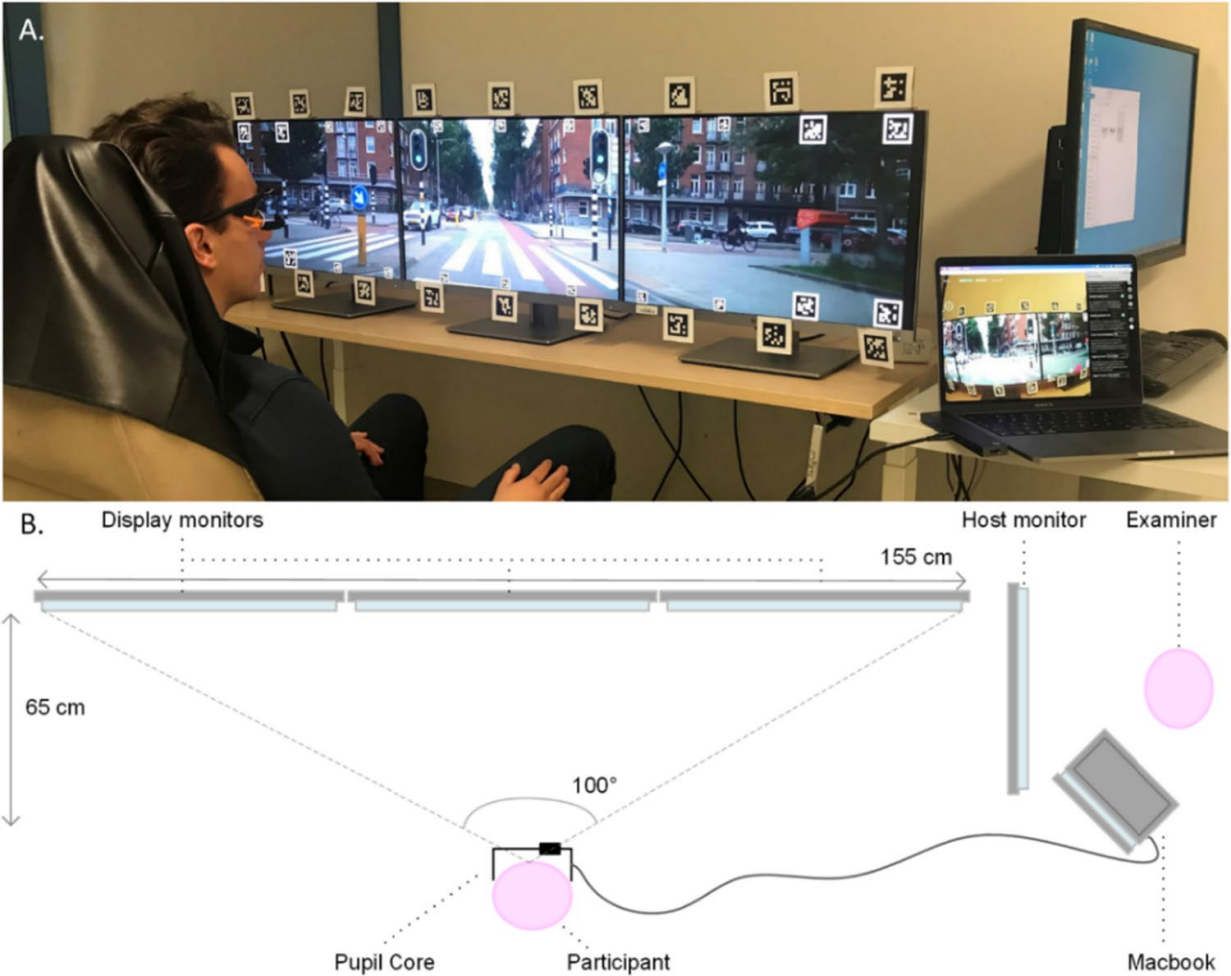
TREYESCAN setup^18^. (**A**) Picture of setup. Participants were seated in front of three display monitors whilst wearing the Pupil Core eye tracker. The individual depicted in the image is not a study participant and has provided consent for the publication of the image. (**B**) Schematic overview of the set-up (view from above). The participant is located 65 cm in front of the central display monitor. The examiner is located on the right of the participant and can guide the experiment from the host monitor. On the MacBook display stability of the signal and performance of the participant could be monitored.

The present study employed an experimental setup and methodology that we described in a previously published work^18^. The experiment took place at Amsterdam University Medical Centers (UMC), location VU University Medical Center (VUmc), and utilized three 24-inch HP EliteDisplay E243i monitors with a resolution of 1920 × 1200 pixels and a pixel density of 94.34 ppi. Participants sat in a car seat positioned 65 cm away from the screens to achieve a 100° field of view, while the table could be adjusted in height to ensure that the eyes were centered on the middle of the screen (Fig. 1). Head movements were permitted in all directions during the experiment.

The participants’ eye movements were recorded by a head-mounted eye tracking device (Pupil Labs Core glasses, received October 2021, Pupil Labs, Berlin, Germany)^19^. The Surface Tracker plugin by Pupil Labs was used to define the surface area of the display with apriltags, which are QR-like markers^20^. This allows the gaze to be mapped on the screen surface, thus obtaining screen-based gaze coordinates with a head-mounted eye tracker.

The data were exported using Pupil Labs Player v3.3.0, and dynamic area of interest analyses were performed using the TREYESCAN Toolkit software^18,21^, written in Python 3.8.3^22^ using NumPy^23^, Pandas^24^, OpenCV^25^, Matplotlib^26^, and SciPy^27^.

### Traffic scenes

Two different driving routes, each with an approximate duration of 30 min, were collaboratively designed with the CBR (Dutch driving test organization), the central office for driver’s license administration in The Netherlands. These routes were specifically selected in the city of Amsterdam, characterized by its urban environment with narrow streets, thereby presenting complex and challenging traffic scenarios. Each route was driven five times.

**Figure 2:**
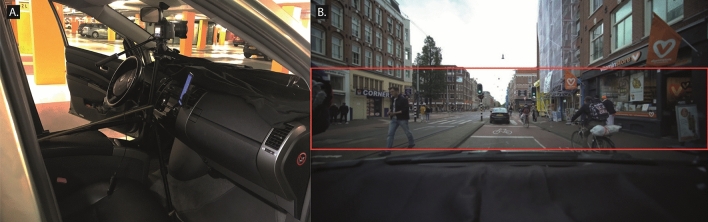
Recording and editing of traffic scenes. (**A**) The car and camera setup. (**B**) Indication of crop to 5760 × 1200. The full width of the video was used. No information about the traffic scene was lost by cropping the video’s height to facilitate screen fitting.

Video footage was captured using a Sony A7III camera equipped with a Laowa Zero-D ultra-wide field 12 mm f/2.8 lens (angle of view: 121.96°; minimal distortion) from within a moving Toyota Prius II vehicle. The camera was mounted centrally behind the windshield, and a black piece of felt was placed on the dashboard to prevent reflections (Fig. 2A). The footage was captured in 4 K resolution (3840 × 2160) at 25 frames per second. The video clips were subsequently expanded and cropped to a resolution of 5760 × 1200 using Adobe Premiere Pro (Adobe Inc, San Jose, CA, USA) to fit the three-screen setup (Fig. 2B).

The video content was analyzed to identify relevant traffic scenarios, that require the driver’s attention, while also ensuring that objects were included from diverse directions in the peripheral visual field. To minimize memory recollection effects, for each traffic scene, a duplicate scene was chosen that contained the same part of the route driven, but with different objects. Finally, a total of 42 traffic scenes were selected and compiled into 6 videos of approximately 8 min each.

### AOIs

In order to determine which objects were relevant to the analysis, a panel of 10 raters comprising 5 experts on practical fitness to drive of the CBR and 5 experienced drivers with knowledge of conducting traffic-related research and/or patient assessment (co-authors RvN, GvR, BM, JK, and LvR), were presented with the 6 videos. The panel had 42.5 (median, IQR [34.0–43.8]) years of driving experience. A web application was developed to enable raters to individually indicate dynamic AOIs in the video by mouse clicks (the source code for this application is available as open-source code on Github^28^: https://github.com/treyescan/marking-aois). The application included options to view in different speed settings, play/pause and rewind the videos. The raters had to choose between two categories, Must-Be-Seen and May-Be-Seen objects. The former included objects that necessitate active or passive consideration by the driver, such as reducing speed, changing lanes and delaying acceleration, while the latter included objects that are relevant to be seen by a driver but do not require a change in driving behavior, such as pedestrians on the sidewalk and oncoming traffic in the other lane.

**Figure 3:**
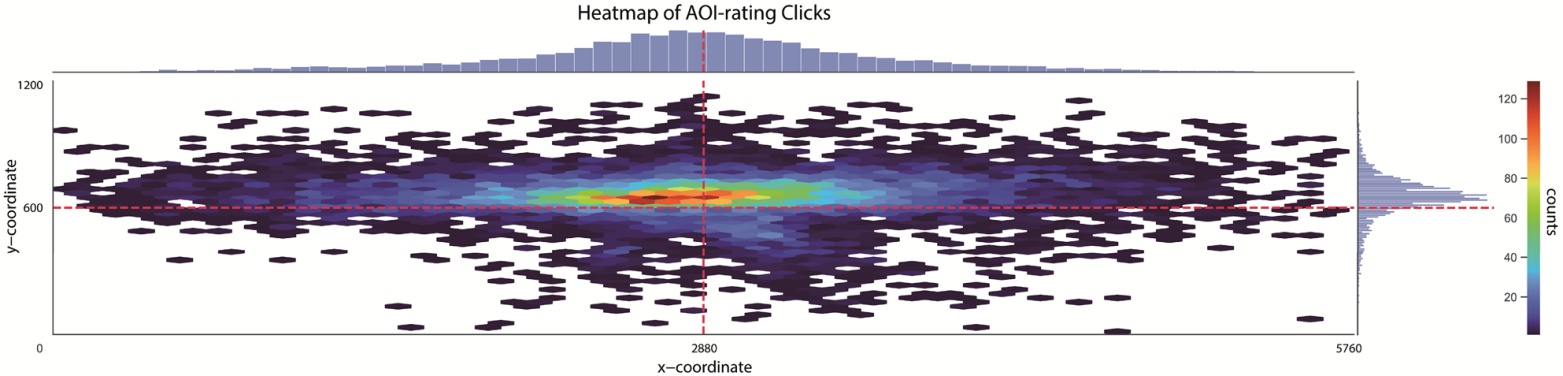
Heatmap of the distribution of all mouse clicks made by the 10 raters for the 6 videos. Dimensions of heatmap represent the dimensions of the video screen. Histograms of distributions of amount of clicks are shown for the x- and y-axis.

Figure 3 presents a heatmap of the distribution of all mouse clicks of the 10 raters for the 6 videos. Notably, most clicks were made in the central part of the screen. The results of the 10 raters were manually coded for each scene in the six videos by observing the videos with an overlay of circle animations representing the location of the clicks. An object was included as a relevant AOI if it was rated by 5 or more raters. If an object was chosen by 4 raters, a panel reviewed its inclusion. The majority of raters determined whether an object was Must-Be-Seen or May-Be-Seen. Traffic lights were included in all cases. A new AOI was incorporated when there was a change in the color of the traffic lights or when the brake lights of a car were activated.

The Dynamic allocation of AOIs was performed using tools that were previously described in our published work^18^. These tools enabled us to determine the location of each AOI for every frame of the video, tracking them from their initial appearance to their disappearance. The TREYESCAN software allows for the addition of margins around the areas of interests, in order to compensate for the inaccuracy of the eye tracker. A margin size of 2.5° was chosen for the analysis.

### Measurements in normal-sighted individuals

The recruitment of normally-sighted participants was done through snowball sampling. Eligibility criteria required participants to have no history of ophthalmic comorbidities and at least 5 years of driving experience. The participants performed an Esterman visual field test^29^ and a visual acuity measurement using the ETDRS chart^30^. Additionally, a customized central visual field test was programmed using the Humphrey Field Analyzer II (HFA). The test employed a three-zone strategy, with an age-corrected test mode according to HFA routines, a III white stimulus, 80 points, and a point spacing of 2º. Its purpose was to screen the central 10° binocularly for any potential visual field defects, as the Esterman visual field test does not measure this area. Only participants with a minimal visual acuity of 0.0 LogMAR (20/20 Snellen or 1.0 decimal notation) and no defects on the visual field tests were included in the study. Participants with multifocal prescription glasses were excluded.

During the experiment, the participants’ eye movements were recorded while they viewed the six videos with traffic scenarios. Following the viewing of three videos, a scheduled intermission was provided, allowing participants to take a break. During this intermission, participants were asked if they had any feedback on the setup, videos, or their experience during the test. The researcher conducting the experiment documented any feedback provided verbally. After all videos had been viewed, the same question was asked to ascertain if participants had supplementary insights or comments contributing to their previously offered feedback. To isolate the visual aspect of the task, the videos were presented without accompanying traffic sounds. The participants were instructed to imagine themselves as the driver of the vehicle and to press a button whenever they identified an object that required action, such as reducing speed or changing lanes. This instruction was given to avoid the interference caused by providing a verbal commentary during concurrent hazard perception tasks^31^. Before each video, a nine-point screen calibration was conducted, followed by a 12-point validation routine, which both extended across the entire screen^18^. The Pupil Labs software then generates a value for the accuracy and precision of the calibration. During the calibration process, we set a target accuracy threshold of less than 2.5º, in line with the specifications provided in the Pupil Labs documentation, which indicates that 3D Gaze Mapping should achieve an accuracy range of 1.5º to 2.5º. When the initial calibration did not meet this accuracy requirement, two additional calibration attempts were performed. After the third calibration, the accuracy value obtained was accepted, also when it exceeded 2.5º.

Following a four-week interval, the participants underwent a second viewing of the videos in a different order to gather data from two separate instances. A randomization tool was employed to determine the viewing order, which could be presented as either 123–456 or 456–123. During the second measurement session, the participant was presented with the opposite viewing order from their initial session. The use of this time interval, coupled with the inclusion of duplicate scenes, was intended to reduce potential recall bias.

All procedures involved in collecting and analyzing data from human subjects were conducted in accordance with the ethical standards of the 1964 Declaration of Helsinki and its subsequent amendments. The experimental protocol was officially approved by the Medical Ethical Committee of Amsterdam UMC, location VUmc (METC-Number: 2020.475). All participants provided informed consent prior to participation. The individual depicted in Fig. 1A was not a study participant and has provided informed consent for the publication of the image in an online open access publication.

### Determining the contents of TREYESCAN

The included participants viewed 6 videos of approximately 8 min on two measurement sessions. For the next study stage, a case–control study with glaucoma patients, the objective is to create a test consisting of the most effective scenes. To determine which videos should be included in the final test, a stepwise approach was used, based on the feedback of the included participants and the traffic scene characteristics.

### Determining the relevant AOIs to include in TREYESCAN

The data obtained from this pilot study will be used to determine the most relevant AOIs for inclusion in the subsequent phase of analysis in the case–control study. We hypothesize that the included normally sighted participants with considerable driving experience, have a good understanding of where their visual focus should lie within the traffic scenes. Consequently, it would be inappropriate to evaluate the patient population in later stages using AOIs that are not looked at by normally sighted individuals. To address this, we propose that an AOI should be included if it has been viewed, arbitrarily, by at least half of the participants.

On the other hand, when conducting AOI analyses in eye tracking, it is important to consider entry time—the duration from the onset of the stimulus until the AOI is first viewed^13^. We observed that certain AOIs do not require a deliberate saccade movement to be immediately “hit”, due to a (close to) central location or added margins. Consequently, it would be unfair to compare entry times for objects in the case–control study that are consistently and falsely “hit” by most participants in this pilot study. To address this issue, we decided to exclude AOIs that start in the central 10º of the screen. Our findings indicate that AOIs originating within the central 10º are predominantly objects in the oncoming lane, which start at a small size and therefore obtaining large margins. Consequently, these AOIs do not capture deliberate gaze behavior since they are immediately and falsely “hit” when looking at the road ahead.

Similarly, we have also encountered other objects starting within the central screen of the setup that exhibit similar characteristics. For the object appearing between the central 10º and 30º, we have introduced an additional variable, namely entry time. We identified AOIs with a short entry time, taking into account previous research indicating that a natural saccade latency of 120–150 ms occurs with everyday stimuli and environments. Hence, we set a threshold of 120 ms for the median entry time of all participants, enabling us to select only deliberate saccades to these AOIs^13,32,33^. The use of median entry time was necessary due to the skewed distribution of entry times. If an object appeared within the central 10º to 30º and had an entry time shorter than 120 ms it would be marked for exclusion in that measurement session (T1 or T2).

In summary, the exclusion of an AOI was determined based on the following criteria for each for each measurement session separately: (1) if it was observed by less than half of the participants, (2) if its appearance was within the central 10º, and (3) if its appearance was within the central 10°–30° and the median entry time was shorter than 120 ms. If any of these criteria were met, the AOI was considered for exclusion in that particular measurement session. Only when an AOI satisfied the exclusion criteria in both measurement sessions, it was generally marked for exclusion.

## Results

### Participant characteristics

A total of twenty participants, with a median age of 34.0 years (IQR [26.0–56.0]), were included in the study. These individuals viewed the six videos with traffic scenes on two measurement sessions separated by a four-week interval. Logistical constraints prevented one participant from participating in the second measurement session.

Table 1 presents the participant characteristics and measurement details. All participants had a minimum of five years of driving experience, showing a wide range of experience, distance traveled, and frequency of days driven per month. The randomization process ensured an equal distribution of the viewing order among participants. None of the participants had any visual impairments, and none self-reported the use of medication that could affect responsiveness and concentration.

**Table 1:** Participant characteristics and measurement details.

	*n* = 20
Age (year) median [IQR]	34.0 [26.0 to 56.0]
Number of female participants (N)	14
Binocular visual acuity (logMAR) median [IQR]	− 0.1 [− 0.1 to − 0.03]
Esterman visual field abnormalities (N)	0
Prescription eyeglasses (N)	1
Contact lenses (N)	1
Education (years) median [IQR]	16.0 [15.0 to 16.0]
Driving experience (years) median [IQR]	15.0 [7.1 to 36.3]
Distance driven per month (km) median [IQR]	200.0 [100.0 to 837.5]
Days driven per month (days) median [IQR]	10.0 [3.3 to 20.0]
Accident involvement	
- Number of accidents with injury	1
- Number of accidents without injury	2
Video order randomization, first measurement:	
− 123–456	9
− 456–123	11

**Figure 4:**
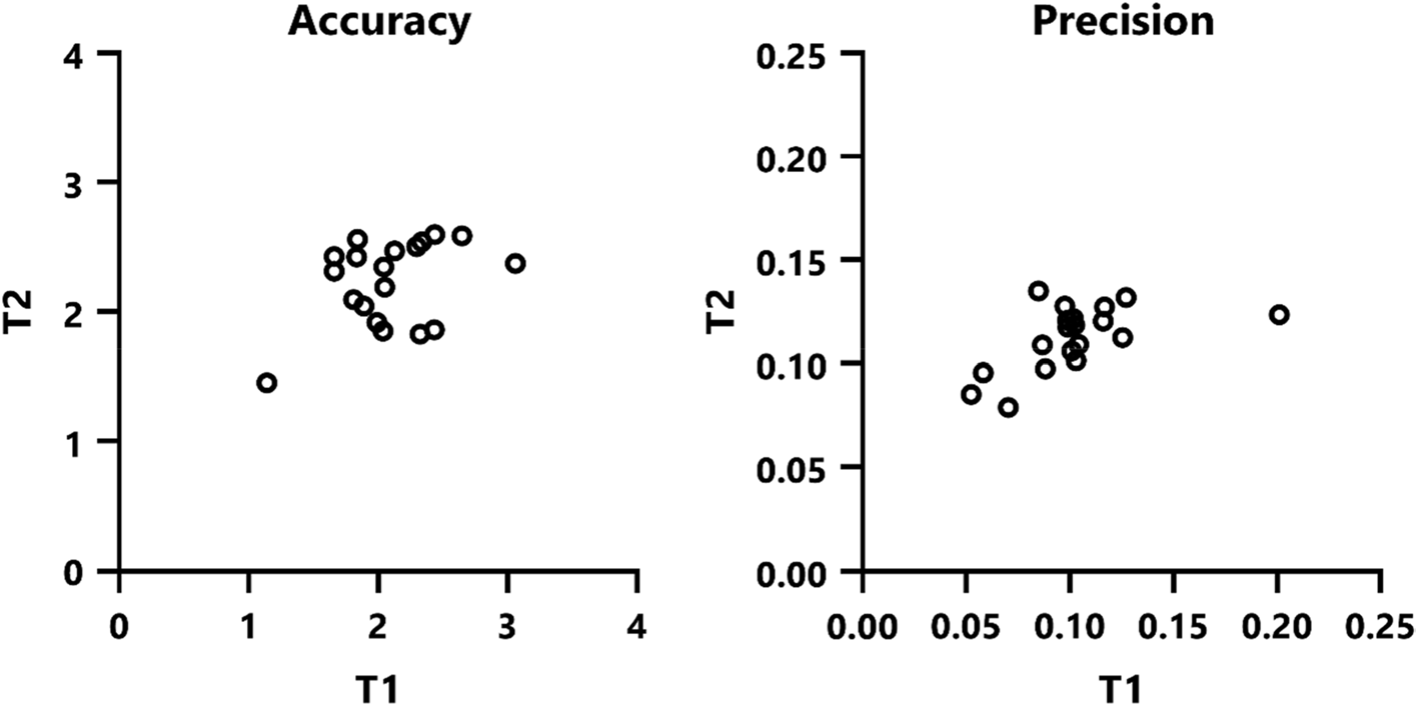
Scatterplots of mean accuracy and precision values as provided by the Pupil Labs validation routine. These measurements were taken prior to the presentation of each video, and the mean values were derived from the six accuracy and precision measurements conducted during a single measurement session.

Figure 4 displays the accuracy and precision measurements derived from the calibration and validation process. The majority of these values lay within the accuracy range specified by Pupil Labs, which is 1.5°–2.5°.

### Screen regions

Table 2 presents the ratios of time spent in different regions of the total screen (three monitors together) to the total video length. Approximately 85% of the total time (0.851 ± 0.106) was dedicated to observing the entire screen, since gaps due to blinking and instances when participants looked away from the screen were not considered as valid gap samples; 85% is considered a reliable outcome for valid gaze samples^13^.

**Table 2:** Ratios of time spent in a region to total video length (mean ± SD).

	All measurements (*n* = 234)	T1 measurements (*n* = 120)	T2 measurements (*n* = 114)
Central 10°*	0.376 ± 0.105	0.378 ± 0.101	0.373 ± 0.109
Central 20°*	0.565 ± 0.116	0.573 ± 0.107	0.557 ± 0.125
Central 30°*	0.675 ± 0.111	0.683 ± 0.099	0.667 ± 0.122
Entire screen	0.851 ± 0.106	0.856 ± 0.086	0.846 ± 0.123
Left side	0.462 ± 0.093	0.474 ± 0.086	0.449 ± 0.098
Right side	0.389 ± 0.072	0.381 ± 0.066	0.398 ± 0.076
Top half	0.223 ± 0.153	0.212 ± 0.156	0.235 ± 0.150
Bottom half	0.628 ± 0.170	0.644 ± 0.175	0.611 ± 0.162

*Diameter is 10°, 20° or 30°.

Furthermore, over 50% of the observation time was focused on the central 20° region of the screen (0.565 ± 0.116). This pattern was consistent across both the T1 and T2 measurement sessions. Additionally, participants tended to spend more time on the left side and the bottom half of the screen. The similarity observed between the T1 and T2 measurements indicates a valid reproducibility of the time spent in specific regions.

### Qualitative participant experience

Following the experiment, participants were asked to provide feedback regarding their experience with the tasks. Participants complimented the realism of the traffic scenes, the setup, and the clarity of the task. Several participants reported experiencing feelings of dizziness or nausea during the tasks, particularly at scenes with unexpected fast turns or when moving over speed bumps. The symptoms of simulator sickness did not prevent participants to finish watching all videos within a single measurement session. Another limitation that participants highlighted was the absence of side- and rearview mirrors, especially when objects came into view from behind the vehicle, resulting in a startled sensation. Additionally, participants reported challenges in estimating the speed and direction of the vehicle, due to the absence of provided information during the tasks and their role as passive observers rather than actively driving. Furthermore, participants expressed that the duration of the task, which involved watching six videos of eight minutes, was quite tiring.

### Qualitative filtering

**Figure 5:**
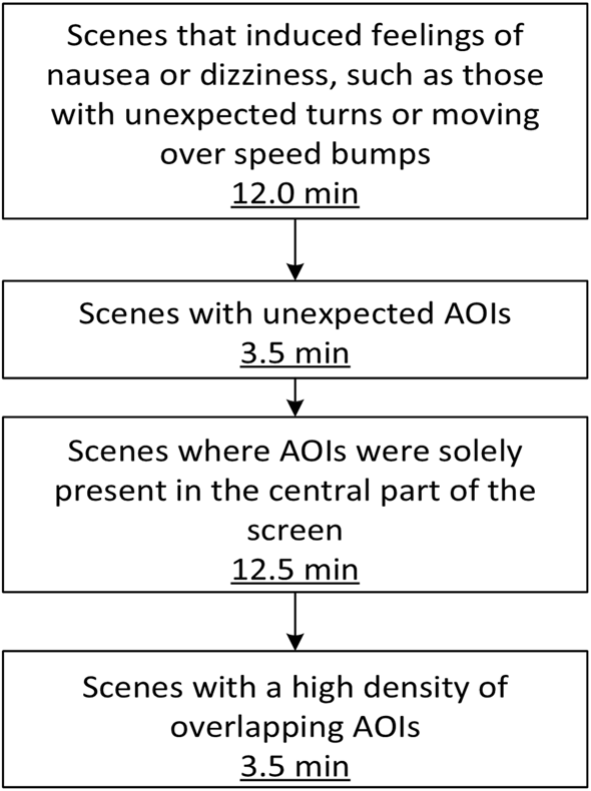
Excluded (parts of) Scenes with Duration of Removal. (1) Parts of scenes with turns and speed bumps were entirely removed. In cases where only the initial or final segment of a scene featured a turn, that specific portion was removed, while the remainder of the scene was retained for subsequent analysis. (2) Scenes or portions were excluded if they included objects entering the view from behind the vehicle. Such instances could provoke a startled sensation among participants, as these objects were not anticipated through the use of side mirrors. (3) After implementing the above-mentioned exclusion steps, certain scenes no longer contained any peripheral AOIs and were confined solely to the central region of the screen. These scenes were subsequently removed from the analysis due to the limited potential for measuring compensatory eye movements. (4) Certain scenes exhibited a high density of relevant AOIs intersecting with one another. To simplify the subsequent AOI analysis, these scenes or parts of scenes were excluded.

Based on the participants’ feedback and their reported experiences, we made the decision to select the most suitable scenes for the final TREYESCAN tasks (two videos of approximately eight minutes). The selection process took into account both participant feedback and specific characteristics of the scenes. The scenes were chosen in the following order, as described in Fig. 5.

### AOI selection

Subsequently, the AOIs within the scenes that remained in the TREYESCAN were further analyzed. The first column in Table 3 displays the distribution of the 325 AOIs across different categories. Out of these, 165 of these AOIs were classified as Must-Be-Seen objects, while 160 were categorized as May-Be-Seen objects.

**Table 3:** Selection of AOIs.

	AOIs before selection	AOI < 10°	N < 10		AOI 10°–30° & ET < 120 ms		Excluded AOIs after selection		
			T1	T2	T1	T2	T1	T2	T1 & T2
Ambulance*	1	0	0	0	1	1	1	1	1
Barricade	1	1	0	0	0	0	1	1	1
Bus	2	1	0	0	0	0	1	1	1
Car	69	43	3	4	10	8	56	55	54
Cyclist	85	28	7	7	14	10	48	45	42
Motorcycle	16	6	0	0	3	4	9	10	9
Pedestrian	85	33	7	5	20	14	57	52	49
Police*	2	1	0	0	0	0	1	1	1
Sign	6	3	0	0	1	0	4	3	3
Traffic light	37	25	1	0	3	5	28	30	27
Tram	4	1	0	0	1	2	2	3	2
Truck	4	2	0	0	0	0	2	2	2
Van	13	8	0	0	3	2	11	10	9
Total AOIs	325	152	18	16	56	46	221	214	201

*Ambulance and police vehicles did not have emergency vehicle signals activated.

AOI < 10°: AOIs that start within central 10º.

N < 10: AOIs that have been viewed by less than 10 participants.

AOI 10°–30° & ET < 120 ms: AOIs that start within central 10° to 30° and median entry time is < 120 ms.

**Figure 6:**
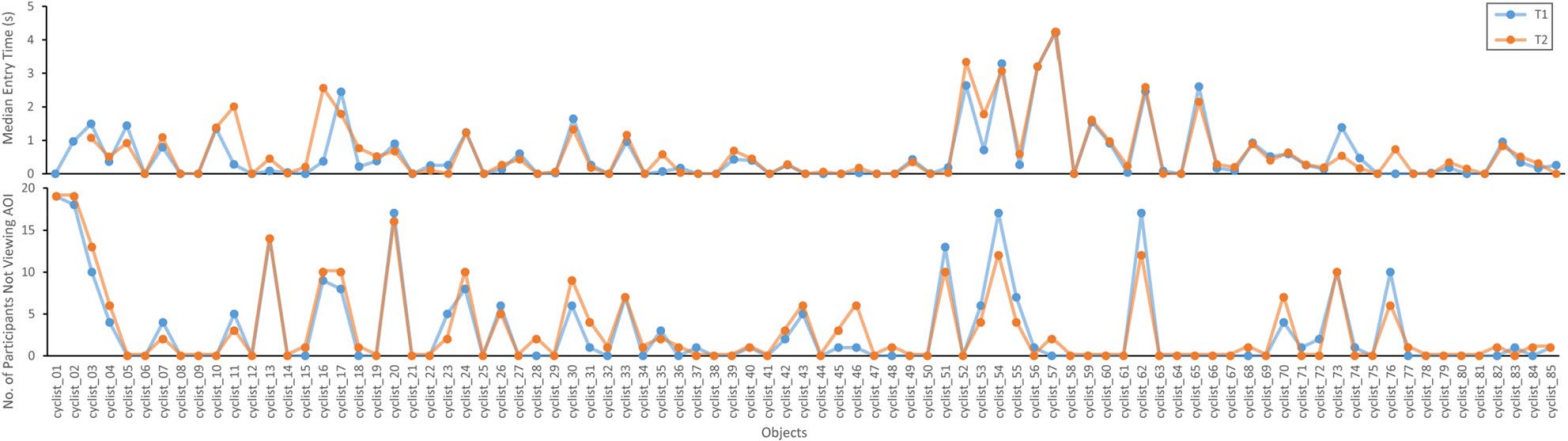
(**A**) Graph representing the median time to first entry for the T1 (Blue) and T2 (Orange) measurements for all the cyclist objects. (**B**) Graph representing the number of participants not viewing an AOI for all the cyclists for the T1 and T2 measurements.

Figure 6 illustrates the median entry time and the number of participants who did not view an AOI for all cyclists objects during the T1 and T2 measurements, as an example. Notably, median entry times for each object are similar for the T1 and T2 measurement sessions, indicating consistent results.

The remaining AOIs were analyzed based on the number of participants who viewed them, their location on the screen, and the entry time, as described in the methods section. Table 3 provides an overview of the exclusion process and portrays the number of AOIs that would be excluded based on each criterion. Notably, while several car AOIs are excluded, a higher proportion of cyclist and pedestrian AOIs remain in the analysis. By applying these exclusion criteria, a total of 124 AOIs out of the initially marked 325 AOIs would remain in the analysis in further phases. Among these 124 remaining AOIs, 64 are categorized as Must-Be-Seen objects and 60 as May-Be-Seen objects.

### Video examples

In the supplementary material S1 (Video Cyclist_6), S2 (Video Cyclist_7), and S3 (Video Cyclist_54), three videos of the task are provided with the gaze data and AOIs with margins of 2.5˚. Video S1_Cyclist_6 serves as an example of an AOI that was excluded based on a short median entry time. Due to the margins and central appearance of the AOI, the entry time was marked as 0 for many participants (median entry time T1: 0 s, median entry time T2: 0 s).

Video S2_Cyclist_7 is an illustrative example of a relevant AOI since the median entry time accurately reflects a deliberate gaze movement towards it (median entry time T1: 0.795 s, median entry time T2: 1.09). Lastly, video S3_Cyclist_54, represents an AOI located on the far right of the video with minimal participant’s gaze towards it (T1: 3 participants, T2: 7 participants). Consequently, it may be justifiable to exclude the marked AOIs in the analysis of the case–control study involving glaucoma patients.

## Discussion

The development of the TREYESCAN may initiate a step forward in understanding compensatory eye movements in glaucoma patients during real-world tasks like driving. By employing a wide field of view and incorporating dynamic areas of interest within traffic scenes, we expect that our eye tracking test might address the limitations of traditional visual field testing methods and might provide valuable insights into the visual exploration and hazard perception abilities of individuals with visual field defects. To improve the test, we utilized data obtained from normally sighted individuals to select the most suitable scenes and AOIs for the final version of the TREYESCAN, which will be employed in a case–control study involving glaucoma patients.

Based on participant feedback, scenes featuring rapid turns and high speed bumps were excluded from the TREYESCAN, due to reports of dizziness or a nauseous feeling. However, none of the participants had to discontinue the measurements due to simulator sickness. In a study conducted by Keshavarz, et al.^34^, it was found that older adults (age 65 +) experienced more simulator sickness than younger adults (age 18–39). It should be noted that the population in our study was relatively young (median age 34.0 years (IQR [26.0–56.0])). In a subsequent phase of the research, which will involve a group of glaucoma patients and age-matched control subjects, it is expected that the participants will be older than this initial group. Therefore, attention must be given to the experiences of this older group concerning simulator-induced sickness.

In our sample, it was observed that participants directed their gaze towards the central 20º of the screen for more than 50% of the total viewing time (0.565 ± 0.116). This remained consistent across both measurement sessions, indicating the absence of a learning effect. A similar finding was reported in a study conducted by Deng, et al.^35^, where they found that drivers predominantly focused their attention on the end of the road in front of the vehicle when viewing traffic images. Similarly, Underwood, et al.^36^ found that both novice and experienced drivers predominantly fixated on the road far ahead and mid ahead while driving a car. Furthermore, our results revealed that participants allocated more viewing time to the left side of the screen (0.462 ± 0.093) compared to the right side (0.389 ± 0.072), and this pattern also remained consistent across both measurement sessions. As can be seen in Fig. 1, the scenes have been recorded in the center of the front windshield, but as we realized in a later stadium: unfortunately slightly skewed to the left. This was the case for one of the two routes driven, of which most scenes were included in the 2 final videos. Figure 3 also shows that more AOIs were selected on the left side of the center of the screen. Consequently, resulting in an increased gaze allocation towards the left side of the screen, when focusing on the road ahead. This is a limitation of our traffic scenes that should be taken into consideration when interpreting future results of the case–control study.

In addition, we examined the relevant AOIs that would be suitable for incorporation into the TREYESCAN analysis for the subsequent phase of the case–control study. Prior research by Smith and Mital^37^, has demonstrated that initial saccades tend to prioritize fixation on the central region of the screen, regions characterized by high motion and people. It is important to acknowledge that this default gaze pattern may have influenced the participants’ visual attention towards the AOIs in our study. It will be interesting to compare the findings in this study with the outcomes derived from the glaucoma patients in the subsequent phase of our research.

The TREYESCAN test demonstrates several strengths that contribute to its utility in assessing compensatory eye movements in individuals with visual field defects, such as glaucoma. By capturing the complexity of real-world driving situations, the test may provide a means to explore how these individuals visually interact with dynamic traffic scenes. The inclusion of a wide field of view and unrestricted head movements addresses visual field defects. This enables participants to utilize their remaining visual field effectively and to explore the traffic scenes naturally. Moreover, the collaboration with experts of practical driving ability and experienced drivers in the development of the test enhances its validity and real-world relevance. Additionally, the pilot measurements conducted with normally sighted individuals establish a valuable baseline, providing a selection for relevant scenes and AOIs, and a reference point for future studies involving glaucoma patients and control subjects.

However, several limitations should be considered. Firstly, simulator sickness experienced by some participants during the test poses a potential challenge that needs to be further investigated in future studies. The absence of side mirrors, rearview mirror and dashboard in the experimental setup may limit the assessment of visual scanning behavior during driving, which may not completely capture the strategies employed by individuals with visual field defects in real-world scenarios. Additionally, the assessment of potential hazards may be influenced by challenges in accurately estimating vehicle speed and direction, arising from the passive role of observing rather than actively driving.

Moving forward, the TREYESCAN will be applied in a case–control study including glaucoma patients and controls. By comparing the eye movements and gaze patterns of these two groups, we aim to study the compensatory mechanisms employed by individuals with glaucoma. The valuable insights obtained from individuals without a visual impairment in this study, have significantly contributed to refining the test by identifying the most effective scenes and AOIs. Furthermore, these insights have established a baseline understanding of the test’s functionality and performance.

## Conclusion

This study presents the development and methodology of our novel eye tracking test, the TREYESCAN. We provide insights into the process of traffic scene selection and the identification of AOIs within these dynamic traffic scenarios by an expert rater panel. Pilot measurements conducted on normally sighted individuals have provided valuable insights into the setup’s performance and serve as a baseline for understanding gaze behavior and eye movements in the dynamic scenes presented. These results contributed to the selection of relevant AOIs to include in the test. Looking ahead, this research project aims to contribute to a better understanding of how individuals with visual field defects utilize compensatory eye movements to overcome visual limitations, enabling them to maintain functional performance in visually demanding situations such as driving.

### Supplementary Information

Supplementary Information 1.

Supplementary Video 1.

Supplementary Video 2.

Supplementary Video 3.

## Data Availability

The raw eye tracking data is made available in an online depository (https://doi.org/10.17026/dans-xes-wdbf). This data can be used in combination with the Dynamic AOI Toolkit.

## Code availability

The source code for the entire toolkit is available on GitHub (https://github.com/treyescan/dynamic-aoi-toolkit) licensed under GNU General Public License version 3.0. The source code for marking Areas of Interest is also available on GitHub (https://github.com/treyescan/marking-aois) licensed under GNU General Public License version 3.0.
