# Supplementary material for: TREYESCAN: configuration of an eye tracking test for the measurement of compensatory eye movements in patients with visual field defects
Source: Sci Rep. 2023 Nov 22;13:20479. doi: 10.1038/s41598-023-47470-5 (PMC10665380; doi:10.1038/s41598-023-47470-5)
Supplement: Supplementary file 1 — Supplementary Information 1. [file 41598_2023_47470_MOESM1_ESM.pdf]

TREYESCAN: Configuration of an Eye Tracking Test for the Measurement of Compensatory Eye Movements in Patients with Visual Field Defects

Yasmin Faraji, MD; Joris W. van Rijn, BSc; Ruth MA van Nispen, PhD; Ger HMB van Rens, MD, PhD; Bart JM Melis-Dankers, PhD; Jan Koopman, PhD; Laurentius J (René) van Rijn, MD, PhD

Supplementary Materials

S1-S3 Video Examples

Every colored dot represents the gaze of one participant in the first measurement session. Labels with participant id are presented next to the dots. Must-Be-Seen AOIs are depicted as pink/red and May-Be-Seen as light blue/dark blue. Margins of 1.5 degrees are depicted around the AOIs as red or dark blue.

S1 Video Cyclist_6

S1_Video_Cyclist6.mp4 is included as a separate file.

S2 Video Cyclist_7

S2_Video_Cyclist7.mp4 is included as a separate file.

S3 Video Cyclist_54

S3_Video_Cyclist54.mp4 is included as a separate file.
